# Universal conservation law governing the wave–particle duality and quantum entanglement

**DOI:** 10.1038/s41377-025-01804-2

**Published:** 2025-03-27

**Authors:** Kai Sun, Jin-Shi Xu, Chuan-Feng Li

**Affiliations:** 1 CAS Key Laboratory of Quantum Information, University of Science and Technology of China, Hefei, 230026 China; 2 CAS Center for Excellence in Quantum Information and Quantum Physics, University of Science and Technology of China, Hefei, 230026 China; 3 Anhui Province Key Laboratory of Quantum Network, University of Science and Technology of China, Hefei, 230026 Anhui China; 4 Hefei National Laboratory, University of Science and Technology of China, Hefei, 230088 China

**Keywords:** Physics, Quantum optics

## Abstract

Universal conservation laws of wave–particle–entanglement triad, which describe relations between the wave–particle duality of a quantum system and its entanglement with an ancilla quantum memory, are proposed and further demonstrated with silicon-integrated nanophotonic chips.

The wave–particle duality of light represents a fundamental concept in quantum physics, describing light’s ability to exhibit both wave-like and particle-like properties depending on the experimental conditions. This duality, however, has long been a subject of debated whether light’s nature is predominantly wave-like or particle-like^1^. A milestone in this field was Niels Bohr’s formulation of the complementary principle^2,3^, which asserts that wave and particle characteristics are mutually exclusive but complementary aspects of light’s nature, with only one aspect observable in any given experimental configuration.

However, Bohr’s complementary principle has been challenged by the groundbreaking delayed-choice experiment, initially proposed by J. Wheeler in his seminal gedankenexperiment^4^. This challenge has been particularly amplified by recent achievements in its quantum version^5^. In 2012, a significant breakthrough occurred when several research groups independently realized the quantum version of Wheeler’s delayed-choice experiment, nearly simultaneously. These experiments demonstrated the quantum superposition of the particle and wave properties of a single photon^6–8^.

Building upon these developments and drawing inspiration from the concept of “Quantum Cheshire Cats” introduced by Yakir Aharonov and his colleagues^9^, which demonstrates the separation of physical properties from their carriers via weak measurement techniques, the wave and particle properties of a single photon can be separated and detected simultaneously in an experiment setup^10,11^. These outstanding works, involving the measurement of complementary phenomena in a single measurement setup, have prompted a profound reinterpretation of the complementarity principle in quantum mechanics.

When extended to more complex quantum systems, the situation becomes more intriguing. The wave–particle duality maintains profound connections with fundamental quantum concepts, particularly the uncertainty principle and quantum entanglement^12,13^, significantly enriching both its theoretical implications and practical applications. A recent work published in *Light*: *Science & Applications*, has made significant strides in demonstration of universal conservation laws for the wave–particle duality and entanglement, conceptualized as the wave–particle–entanglement triad, in both theory and experiment^14^. With the help of an ancilla quantum memory, this work introduces a theoretical framework that quantitatively correlates wave-like property, particle-like property, and entanglement, extending these relationships to high-dimensional quantum systems. The experimental validation of these conservation laws was achieved using silicon-integrated nanophotonic chips, enabling precise quantification and comparative analysis of these three fundamental quantum properties within a controlled platform.

As illustrated in Fig. 1, Ding et al. demonstrated that the wave–particle–entanglement triad adheres to a fundamental conservation law^14^. Through rigorous quantification of the three elements within their respective operational frameworks, the researchers established that the sum of these quantified parameters remains constant, with this invariant being intrinsically determined by the system’s dimensionality. In their experiment, an advanced setup with the silicon-integrated nanophotonic quantum chip enables to precisely control and adjust the weights of particle and wave behaviors. They quantify the three elements across divers operational frameworks, including the resource language of entropy and the framework that employs path predictability as the metric for particle-like behavior, interference visibility of paths in the chip for wave-like characteristics, and concurrence for entanglement quantification. Supported by robust experimental results, they successfully verified the conservation laws within various experimental configurations and demonstrated that the conservation constant decreases with the increasing system noise.

**Fig. 1:**
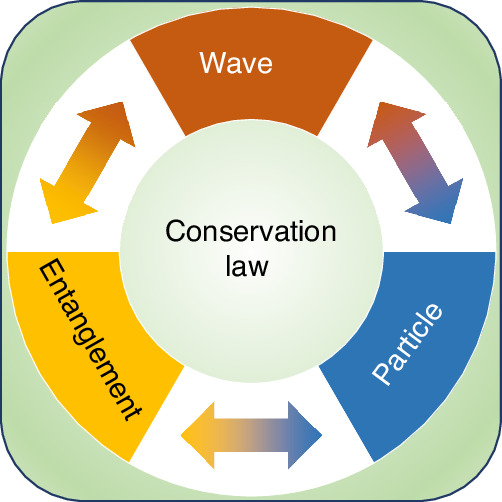
Illustration of the conservation law for the wave–particle–entanglement triad. The diagram illustrates that the sum of the quantified measures of these three properties is constrained by a fundamental constant. Through the conservation law, it is possible that the relative weights of wave-like behavior, particle-like behavior, and entanglement can be dynamically transformed within quantum systems

This work offers a novel perspective on the wave–particle duality and quantum entanglement through the lens of quantum resource theory, providing a comprehensive framework to investigate the intricate interplay between these fundamental quantum phenomena. It also advances the understanding of duality in multipartite systems. The exquisite experiment also extends the ability to investigate fundamental quantum physics on on-chip quantum platform. Based on the conservation law governing the wave–particle–entanglement triad, it enables the precise manipulation and transformation of these properties within quantum systems. Beyond its profound implications for quantum fundamental theory, this work also inspires practical applications of these resources in quantum technologies, including but not limited to the quantum memory^15^, quantum precision measurement^16^, and counterfactual communication^17^.
